# Patterns of Referral for Common Cancer Surgery in the United States

**DOI:** 10.1245/s10434-025-17026-0

**Published:** 2025-02-18

**Authors:** Kelsey B. Montgomery, Elizabeth Ross, Chimaraije Amu-Nnadi, Smita Bhatia, Kristy K. Broman

**Affiliations:** 1 Department of Surgery, University of Alabama at Birmingham, Birmingham, AL USA; 2 Institute for Cancer Outcomes and Survivorship, University of Alabama at Birmingham, Birmingham, AL USA; 3 Rutgers University, New Brunswick, NJ USA; 4 Department of Pediatrics, University of Alabama at Birmingham, Birmingham, AL USA; 5 Department of Veterans Affairs, Birmingham VA Medical Center, Birmingham, AL USA

**Keywords:** Surgical cancer care, Cancer hospitals, Centralization, Referral care, Cancer care delivery

## Abstract

**Background:**

Shifts in healthcare delivery have resulted in most U.S. hospitals participating in integrated health systems, many of which selectively refer complex cancer surgery to high-volume centers. However, this centralization may exacerbate barriers to access and may not be necessary for all cancer types. This study describes the prevalence and pattern of referral for surgery for common cancers and evaluate associated factors.

**Methods:**

The National Cancer Database was used to identify adult patients who underwent curative-intent surgical resection between 2010 and 2020 for 12 common cancers (bladder, breast, colon, kidney, lung, melanoma, oral cavity, pancreas, prostate, rectum, thyroid, and uterus). The primary outcome was receipt of referred surgical cancer care.

**Results:**

Overall, 5,406,813 patients underwent surgical resection for common cancers, with 33.7% referred for surgery after diagnosis elsewhere. Rates of referred surgery varied by disease site, ranging from 13.7% (bladder) to 58.2% (melanoma). On multivariable analysis, patients with melanoma, oral cavity, prostate, rectal, and uterine cancers (referent = breast), higher clinical stages, and increasing year of diagnosis had higher adjusted odds of referred surgical care. Nonacademic facility types, lower facility volume, higher comorbidity burden, and nonprivate insurance were associated with reduced odds of referred surgical care.

**Conclusions:**

Likelihood of referred surgical cancer care increased over time for 11 of 12 common cancers, with the prevalence of referred care varying significantly based on disease site and sociodemographic factors. Future work evaluating associated clinical outcomes will aid in decisions regarding allocation of referral of surgical cancer care within health systems.

**Supplementary Information:**

The online version contains supplementary material available at 10.1245/s10434-025-17026-0.

Location of surgical cancer care often determines which treatments are available to patients and the quality of care they receive and can be heavily influenced by patient and disease factors, such as insurance status or disease complexity. While volume-outcome relationships for complex cancer care have driven centralization of these cases to high-volume medical centers, surgical cancer care at regional centers can be costly for patients and health systems and may not be necessary for all cancer types or stages.^1,2^

Additionally, major shifts in healthcare delivery in the United States have seen the majority of hospitals now participating in integrated health systems. As consolidation of U.S. hospitals into parent health systems continues, the ability to strategically allocate surgical and other multidisciplinary cancer care among facilities within a system will offer a unique opportunity to optimize the delivery of high-quality cancer care, particularly for common cancers that could possibly be offered in more local settings. With known variation in outcomes based on location of care and the ongoing evolution of the U.S. healthcare system toward increasingly integrated health systems, a foundational understanding of the current patterns of referral and nonreferral care cancer care delivery is needed to support future evaluation of the effects of health system integration on distribution of surgical care, cost, and clinical outcomes.

This study describes the patterns of referred surgical cancer care and location of surgery in a contemporary cohort of U.S. patients with common cancers using the National Cancer Database (NCDB). Furthermore, the study evaluated the association between patient and disease characteristics and receipt of referral surgical care.

## Methods

### Study Overview

This study was deemed exempt by the University of Alabama at Birmingham Institutional Review Board. Using data from the American Cancer Society Cancer Facts and Figures 2022, the top 12 disease sites of new solid organ cancer diagnoses in the United States among combined sexes were identified as breast, colon, prostate, bladder, melanoma, kidney, thyroid, lung, oral cavity/pharynx, rectum, uterus, and pancreas.^3^ Using the above list of common cancers, the NCDB 2020 Participant User Files (PUFs) for the respective cancer sites were queried to identify adult patients who underwent surgical resection of one of these 12 common cancers. The NCDB is a collaborative dataset from the American College of Surgeons and the American Cancer Society that collects data for all patients with newly diagnosed cancers who are treated at Commission on Cancer (CoC)-accredited facilities, and captures more than 70% of all new cancer cases in the United States.^4,5^ Strengthening the Reporting of Observational Studies in Epidemiology (STROBE) guidelines were followed.^6^

### Cohort Selection

Adult patients (age ≥18 years at cancer diagnosis) with stage 0 through IV cancers who underwent surgical resection at CoC-accredited facilities between 2010 and 2020 were included in the analytic cohort. The NCI Surveillance, Epidemiology, and End Results (SEER) Registrar Staging Assistant Extent of Disease database was used to determine appropriate histology code ranges for cohort selection within each cancer site.^7^ The primary site codes, histology codes, and NCDB PUFs used to create analytic cohorts are listed in Supplemental Table 1. The American Joint Committee on Cancer (AJCC) 7th edition staging system was used to determine stage for cases in years 2010–2017 and the 8th edition staging system for years 2018–2020.^8,9^ The NCDB codes for surgical procedure of the primary site were used to identify patients who underwent surgical resection of their primary cancer at the reporting facility; palliative and nonextirpative resections were excluded. Cases were also excluded if no surgical treatment was provided at the reporting facility.

**Table 1:** Cohort descriptive statistics

Characteristic	Cohort (*N* = 5,406,813)
Disease site	
Breast	1,764,485 (32.6%)
Colon	549,371 (10.2%)
Prostate	525,461 (9.7%)
Bladder	480,175 (8.9%)
Melanoma	450,657 (8.3%)
Kidney	379,514 (7.0%)
Thyroid	331,129 (6.1%)
Lung	301,963 (5.6%)
Oral cavity	200,049 (3.7%)
Rectum	182,803 (3.4%)
Uterus	174,439 (3.2%)
Pancreas	66,767 (1.2%)
Age (years)	64.0 (55.0, 72.0)
Sex	
Female	3,255,393 (60.2%)
Male	2,151,420 (39.8%)
Race/ethnicity	
Non-Hispanic White	4,243,384 (80.8%)
Non-Hispanic Black	503,595 (9.6%)
Hispanic	290,385 (5.5%)
Asian or Pacific Islander	169,635 (3.2%)
Other or unknown	42,790 (0.8%)
Insurance payor	
Private insurance	2,445,673 (45.2%)
Medicare	2,428,537 (44.9%)
Medicaid	291,217 (5.4%)
Not insured	106,511 (2.0%)
Insurance status unknown	68,150 (1.3%)
Other government	66,725 (1.2%)
Charlson–Deyo comorbidity index	
0	4,081,422 (75.5%)
1	915,224 (16.9%)
2	257,526 (4.8%)
≥ 3	152,641 (2.8%)
AJCC clinical stage^a^	
0	757,585 (16.3%)
I	2,358,441 (50.8%)
II	1,075,243 (23.2%)
III	310,969 (6.7%)
IV	139,914 (3.0%)
Travel distance (miles)	
< 25 miles	3,555,954 (75.8%)
25–100 miles	929,825 (19.8%)
> 100 miles	203,883 (4.3%)
Urban/rural residence status	
Metropolitan	4,508,286 (83.4%)
Urban	652,678 (12.1%)
Unknown	161,515 (3.0%)
Rural	84,334 (1.6%)
Receipt of referral surgical care	
Nonreferred	3,586,609 (66.3%)
Referred	1,820,205 (33.7%)
CoC facility type^b^	
Comprehensive Community Cancer Program	1,935,607 (35.8%)
Academic/Research Program	1,868,133 (34.6%)
Integrated Network Cancer Program	1,042,012 (19.3%)
Community Cancer Program	304,702 (5.6%)
Suppressed	256,359 (4.7%)
Facility volume tertile	
High	3,816,935 (70.6%)
Moderate	1,188,585 (22.0%)
Low	401,280 (7.4%)

Data represented as frequency (%) or median (IQR)

^a^AJCC = American Joint Committee on Cancer; 7th or 8th edition as applicable by year of diagnosis (2010–2017: 7th edition, 2018-2020: 8th edition)

^b^CoC = Commission on Cancer; facility type is suppressed by the National Cancer Database for patients 39 years old or younger

### Study Outcomes and Definitions

The primary outcome was receipt of referred surgical cancer care. To determine referred care, NCDB class of case information was summarized into two groups: patients who received both diagnosis and some or all treatment at the reporting facility (surgical care where diagnosed, “Nonreferred”), and those who received some or all treatment but no diagnosis at the reporting facility (referred for surgery, “Referred”). As patients who did not receive surgical treatment at their reporting facility were excluded, those who received treatment only and were not diagnosed at the reporting facility were considered to have received referred surgical care.

We also examined the association referred surgical care, CoC facility type, and facility volume. Based on CoC-accreditation standards, CoC facilities represented in the NCDB are categorized into four types: Academic/Research Programs (Academic), Comprehensive Community Cancer Programs (CCCP), Integrated Network Cancer Programs (INCP), and Community Cancer Programs (CCP).^10^ Within the NCDB PUFs, NCI-Designated Comprehensive Cancer Programs are included in the Academic facility type, and Veterans Affairs Cancer Programs are excluded due to federal restrictions. Additionally, the NCDB suppresses facility type identification for patients younger than age 40. Facility surgical volume tertiles were created by summing the total number of analytic cases across all disease sites from each facility, then dividing the facilities into three quantiles by total cases per facility such that an equal number of facilities was included in each tertile. Thus, facility surgical volume tertile is a relative categorization for this study and was not tied to specific volume cutoffs.

Using the NCDB variable for area-based measure of rurality (based on U.S. Department of Agriculture Economic Research Survey data and definitions for metropolitan, urban, and rural groupings), “Metro” was defined as counties in metro areas of > 20,000 people, “Urban” included populations of 2500 to 20,000 people, and “Rural” included < 2500 people. A categorical travel distance variable was created by grouping travel distances into three categories: < 25 miles, 25–100 miles, and > 100 miles, which were felt to convey a more descriptive representation of the range of travel burden than a continuous travel distance.

### Statistical Analysis

Descriptive statistics were performed, with values represented as medians with interquartile ranges (IQR) for continuous data, and frequencies with percentages for categorical data. Bivariate analyses to compare patient, disease, and facility characteristics between Nonreferred and Referred groups were conducted using Pearson’s chi-squared tests for categorical data and Kruskal–Wallis rank-sum tests for continuous data. Next, a mixed-effects multivariable logistic regression model was created to evaluate the likelihood of referred surgical care, with facility identifiers as random effects to account for facility-level clustering. Model covariates included year of diagnosis, patient age, race/ethnicity, insurance status, travel distance, metro/urban/rural status, Charlson–Deyo comorbidity index (CDCI), AJCC clinical stage, CoC facility type, and facility volume tertile. Following primary analyses in the entire cohort, disease-specific subanalyses were conducted; disease-specific mixed effects multivariable logistic regression models were created using the same facility-level random effects and model covariates as described above for the combined model. Alpha level for all statistical tests was set at 0.05. Statistical analyses were performed using R version 4.3.1 (R Core Team).^11^

## Results

### Descriptive Statistics

A cohort of 5,406,813 adult patients who underwent surgical resection for the 12 common cancers was identified. Overall cohort descriptive statistics are detailed in Table 1. Nearly one-third of this surgical cohort consisted of patients with breast cancer (1,764,485; 32.6%), with the next most common disease sites being colon (549,371; 10.2%), prostate (525,461; 9.7%), bladder (480,175; 8.9%), and melanoma (450,657; 8.3%). The median age at cancer diagnosis was 64.0 years (interquartile range [IQR] 55.0, 72.0) and 60.2% were female, with variation noted by disease site. The majority of patients were Non-Hispanic White (4,243,384; 80.8%), and most were insured either privately (2,445,673; 45.2%) or by Medicare (2,428,537; 44.9%). The median travel distance to the treating hospital was 10.6 miles (IQR 4.8, 24.0), although nearly one-quarter of this cohort lived 25 miles or farther from their treating facility (1,133,708; 24.1%). The majority of patients lived in a metropolitan area (4,540,167; 83.4%).

**Fig. 1:**
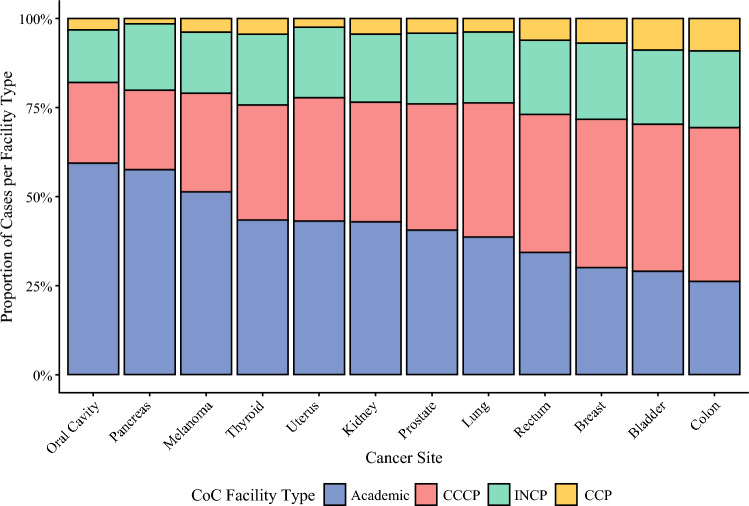
Distribution of Commission on Cancer (CoC) facility type for surgical resection by disease site. Cases with suppressed facility type data (due to age < 40 years) were excluded

There were a total of 1329 unique facilities, of which 485 (36.5%) were CCCPs, 328 (24.7%) were INCPs, 301 (22.6%) were CCPs, and 215 (16.2%) were Academic programs. Most patients underwent surgery at a CCCP (1,935,607; 35.8%) or Academic facility (1,868,133; 34.6%), with smaller proportions at INCP (1,042,012; 19.3%) or CCP (304,702; 5.6%) facilities. The proportional distributions of facility type varied significantly by disease site, with the top five disease sites with the highest proportion of surgical care at Academic facilities being oral cavity, pancreas, melanoma, thyroid, and uterus (Fig. 1).

**Fig. 2:**
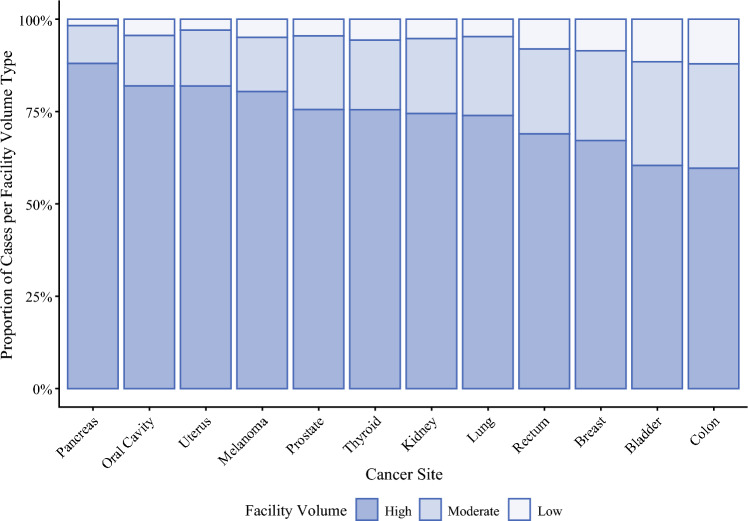
Distribution of facility surgical volume tertile by disease site

One third of facilities (those in the highest volume tertile) treated 70.6% of all patients in this cohort compared with the moderate-volume facilities (22.0% of cohort) and low-volume facilities (7.4% of cohort). Similar to facility type, significant variation in the proportion of cases at each volume tertile by disease site was observed (Fig. 2). Three disease sites had greater than 80% of patients treated at high-volume facilities (melanoma 80.6%, uterus 81.8%, pancreas 88.0%), and all sites had 60% or greater of their respective cohorts treated at high-volume centers. The disease sites with the highest proportions of surgical resection at low-volume facilities were bladder (11.5%) and colon cancers (12.1%).

### Primary Outcome

Two-thirds of the cohort underwent surgery at facilities where they had received their cancer diagnosis (Nonreferred, 66.3%), whereas the remainder received referred surgical care (Referred, 33.7%). Comparison of patient and disease characteristics between Nonreferred and Referred groups are described in Table 2. Compared with the Nonreferred patients, the Referred group had a higher proportion of privately insured patients (49.6% vs. 43.0%), patients with stage II–IV disease (37.6% vs. 30.3%), and patients who traveled between 25 and 100 miles (28.6% vs. 15.4%) or greater than 100 miles (8.3% vs. 2.3%); all comparisons had a *p* value of < 0.001 on bivariate analyses. Referred patients were more commonly treated at an Academic facility (45.2% of Referred cases vs. 29.2% of Nonreferred), and at facilities in the highest volume tertile (80.2% of Referred vs. 65.7% of Nonreferred). Among patients treated at Academic facilities, 56.0% were Referred, compared with 20.6% among patients treated at CCP facilities. There were similar proportions of Nonreferred versus Referred patients among the metropolitan (32.5%), urban (36.3%), and rural cohorts (34.6%), although these differences were statistically significant due to the large cohort size (*p* < 0.001).

**Table 2:** Bivariate analyses of patient and facility characteristics by receipt of referral surgical care

Characteristic	Nonreferred *N* = 3,586,609	Referred *N* = 1,820,205	*p*
Age (years)	65.0 (55.0, 73.0)	63.0 (54.0, 71.0)	< 0.001
Race/ethnicity			
Non-Hispanic White	2,798,751.0 (80.4%)	1,444,633.0 (81.6%)	< 0.001
Non-Hispanic Black	360,062.0 (10.3%)	143,533.0 (8.1%)	
Hispanic	185,302.0 (5.3%)	105,084.0 (5.9%)	
Asian or Pacific Islander	109,378.0 (3.1%)	60,257.0 (3.4%)	
Other or unknown	25,715.0 (0.7%)	17,075.0 (1.0%)	
Sex			
Female	2,187,714.0 (61.0%)	1,067,679.0 (58.7%)	< 0.001
Male	1,398,894.0 (39.0%)	752,526.0 (41.3%)	
Insurance payor			
Private insurance	1,542,052.0 (43.0%)	903,621.0 (49.6%)	< 0.001
Medicare	1,688,718.0 (47.1%)	739,819.0 (40.6%)	
Medicaid	200,417.0 (5.6%)	90,801.0 (5.0%)	
Not insured	78,238.0 (2.2%)	28,273.0 (1.6%)	
Insurance status unknown	39,539.0 (1.1%)	28,611.0 (1.6%)	
Other government	37,645.0 (1.0%)	29,080.0 (1.6%)	
Charlson–Deyo comorbidity index			
0	2,656,018.0 (74.1%)	1,425,405.0 (78.3%)	< 0.001
1	629,066.0 (17.5%)	286,158.0 (15.7%)	
2	186,835.0 (5.2%)	70,691.0 (3.9%)	
≥ 3	114,690.0 (3.2%)	37,951.0 (2.1%)	
AJCC clinical stage			
0	555,659.0 (18.6%)	201,926.0 (12.2%)	< 0.001
I	1,531,236.0 (51.2%)	827,206.0 (50.2%)	
II	634,505.0 (21.2%)	440,738.0 (26.7%)	
III	185,811.0 (6.2%)	125,158.0 (7.6%)	
IV	85,743.0 (2.9%)	54,171.0 (3.3%)	
Travel distance (miles)			
< 25 miles	2,562,117.0 (82.3%)	993,837.0 (63.1%)	< 0.001
25–100 miles	478,430.0 (15.4%)	451,395.0 (28.6%)	
> 100 miles	72,949.0 (2.3%)	130,934.0 (8.3%)	
Urban/rural status			
Metropolitan	3,042,015.0 (86.6%)	1,466,272.0 (84.7%)	< 0.001
Urban	416,079.0 (11.8%)	236,599.0 (13.7%)	
Rural	55,117.0 (1.6%)	29,217.0 (1.7%)	
CoC facility type			
Comprehensive community cancer program	1,418,746.0 (39.6%)	516,861.0 (28.4%)	< 0.001
Academic/research program	1,046,148.0 (29.2%)	821,985.0 (45.2%)	
Integrated network cancer program	718,559.0 (20.0%)	323,454.0 (17.8%)	
Community cancer program	242,021.0 (6.7%)	62,681.0 (3.4%)	
Suppressed	161,135.0 (4.5%)	95,224.0 (5.2%)	
Facility volume tertile			
High	2,357,970.0 (65.7%)	1,458,966.0 (80.2%)	< 0.001
Moderate	908,096.0 (25.3%)	280,489.0 (15.4%)	
Low	320,531.0 (8.9%)	80,749.0 (4.4%)	

Data represented as case frequency (%) or median (IQR); percentages calculated by column

Results from the multivariable analysis are presented in Table 3. Compared with the referent of breast cancer, patients with melanoma (odds ratio [OR] 3.27, confidence interval [CI] 3.24–3.31), oral cavity (OR 1.25, CI 1.23–1.27), prostate (OR 1.36, CI 1.35–1.37), rectum (OR 1.50, CI 1.48–1.52), and uterus (OR 1.89, CI 1.87–1.92) cancers had increased odds of referred surgical care. The remaining disease sites (bladder, colon, kidney, lung, pancreas, and thyroid) had decreased odds of referred surgical care, as detailed in Table 3. The odds of referred surgical care for 2020 were 37% higher compared with 2010, with a generally stepwise increase per year over the course of the study period.

**Table 3:** Mixed-effects multivariable logistic regression model results for likelihood of referred surgical care

Covariate [Referent]	Odds ratio (95% CI)	*p*
Disease site [Breast]		
Bladder	0.35 (0.35–0.36)	< 0.001
Colon	0.77 (0.76–0.78)	< 0.001
Kidney	0.44 (0.44–0.45)	< 0.001
Lung	0.58 (0.57–0.58)	< 0.001
Melanoma	3.27 (3.24–3.31)	< 0.001
Oral cavity	1.25 (1.23–1.27)	< 0.001
Pancreas	0.51 (0.50–0.52)	< 0.001
Prostate	1.36 (1.35–1.37)	< 0.001
Rectum	1.50 (1.48–1.52)	< 0.001
Thyroid	0.47 (0.46–0.48)	< 0.001
Uterus	1.89 (1.87–1.92)	< 0.001
Age (years)	0.99 (0.99–0.99)	< 0.001
CoC facility type [Academic]		
Comprehensive community cancer program	0.65 (0.55–0.77)	< 0.001
Community cancer program	0.70 (0.56–0.87)	0.002
Integrated network cancer program	0.88 (0.73–1.06)	0.2
Facility volume tertile [Highest]		
Moderate	0.64 (0.56–0.74)	< 0.001
Low	0.48 (0.40–0.57)	< 0.001
Race/ethnicity [Non-Hispanic White]		
Asian or Pacific Islander	1.09 (1.08–1.11)	< 0.001
Hispanic	1.04 (1.03–1.05)	< 0.001
Non-Hispanic Black	0.81 (0.81–0.82)	< 0.001
Other or unknown	1.17 (1.14–1.20)	< 0.001
Insurance payor [Private]		
Medicare	0.97 (0.97–0.98)	< 0.001
Medicaid	0.91 (0.89–0.92)	< 0.001
Not insured	0.74 (0.72–0.75)	< 0.001
Other government	1.39 (1.36–1.42)	< 0.001
Unknown	1.09 (1.06–1.12)	< 0.001
Charlson–Deyo comorbidity index [0]		
1	1.00 (1.00–1.01)	0.3
2	0.94 (0.93–0.96)	< 0.001
≥ 3	0.87 (0.86–0.89)	< 0.001
AJCC clinical stage [0]		
I	1.52 (1.51–1.54)	< 0.001
II	1.87 (1.85–1.89)	< 0.001
III	1.89 (1.87–1.92)	< 0.001
IV	1.97 (1.93–2.00)	< 0.001
Travel distance category [< 25 miles]		
25–100 miles	2.44 (2.42–2.45)	< 0.001
> 100 miles	3.98 (3.92–4.04)	< 0.001
Urban/rural residence status [Metropolitan]		
Urban	1.15 (1.14–1.16)	< 0.001
Rural	1.08 (1.06–1.10)	< 0.001
Diagnosis year [2010]		
2011	1.05 (1.04–1.07)	< 0.001
2012	1.11 (1.09–1.12)	< 0.001
2013	1.15 (1.14–1.16)	< 0.001
2014	1.17 (1.16–1.19)	< 0.001
2015	1.21 (1.20–1.23)	< 0.001
2016	1.21 (1.20–1.23)	< 0.001
2017	1.26 (1.24–1.27)	< 0.001
2018	1.33 (1.31–1.34)	< 0.001
2019	1.39 (1.38–1.41)	< 0.001
2020	1.37 (1.35–1.38)	< 0.001

Random effects were created with facility identifiers to account for facility-level clustering; an intraclass correlation coefficient of 0.22 was observed

Patients with higher clinical stage (increase from Stage I OR 1.52 [CI 1.51–1.54] to Stage IV OR 1.97 [CI 1.93–2.00]; referent Stage 0), longer travel distances (OR 2.44 [CI 2.42–2.45] for travel 25–100 miles, OR 3.98 [CI 3.92–4.04] for travel > 100 miles; referent < 25 miles), residence in urban or rural areas (OR 1.15 [CI 1.14–1.16] and OR 1.08 [CI 1.06–1.10], respectively; referent metropolitan), and those of Asian or Pacific Islander or Hispanic race/ethnicity (OR 1.09 [CI 1.08–1.11] and OR 1.04 [CI 1.03–1.04], respectively; referent Non-Hispanic White) also had higher odds of referred surgical care. Likelihood of referred care decreased with increasing CDCI score (CDCI ≥ 3 OR 0.87 [CI 0.86–0.89]; referent CDCI 0), for patients treated at non-Academic facility types (all OR < 1.00 for CCCP, CCP, and INCP facility types; statistically significant for CCCP (*p* < 0.001) and CCP (*p* = 0.002) facilities) or low or moderate-volume facilities (OR 0.48 [CI 0.40–0.57] and OR 0.64 [CI 0.56–0.74] respectively; referent high volume). Additionally, those of Non-Hispanic Black race/ethnicity (OR 0.81 [CI 0.81–0.82]), and patients insured through Medicare or Medicaid and those not insured (OR range 0.74–0.91), also had reduced odds of referred surgical care. The intraclass correlation coefficient (ICC), which represents the proportion of variation in the outcome of interest (i.e., referred surgical care) attributable to clustering (i.e., facility-level effects), was 0.22 for this multivariable model.

### Disease-Specific Subanalyses

The proportion of Referred versus Nonreferred cases varied widely across disease sites (Table 4). Five of 12 sites had more than 40% of cases referred for surgical treatment, including melanoma (58.2%), uterus (52.4%), prostate (47.1%), oral cavity (47.0%), and rectal (43.1%) cancers.

**Table 4:** Proportion of cases per disease site who were Referred or Nonreferred for surgery. Disease sites listed in descending order of referral care; percentages calculated by row (i.e., disease site)

Disease site	Nonreferred *N* = 3,586,609	Referred *N* = 1,820,205
Melanoma	188,208 (41.8%)	262,449 (58.2%)
Uterus	86,965 (47.6%)	95,838 (52.4%)
Prostate	277,990 (52.9%)	247,471 (47.1%)
Oral cavity	92,521 (53.0%)	81,919 (47.0%)
Rectum	113,775 (56.9%)	86,274 (43.1%)
Breast	1,190,991 (67.5%)	573,494 (32.5%)
Pancreas	46,471 (69.6%)	20,296 (30.4%)
Lung	220,449 (73.0%)	81,514 (27.0%)
Colon	403,952 (73.5%)	145,419 (26.5%)
Kidney	293,675 (77.4%)	85,839 (22.6%)
Thyroid	257,339 (77.7%)	73,790 (22.3%)
Bladder	414,273 (86.3%)	65,902 (13.7%)
Total	3,586,609 (66.3%)	1,820,205 (33.7%)

**Fig. 3:**
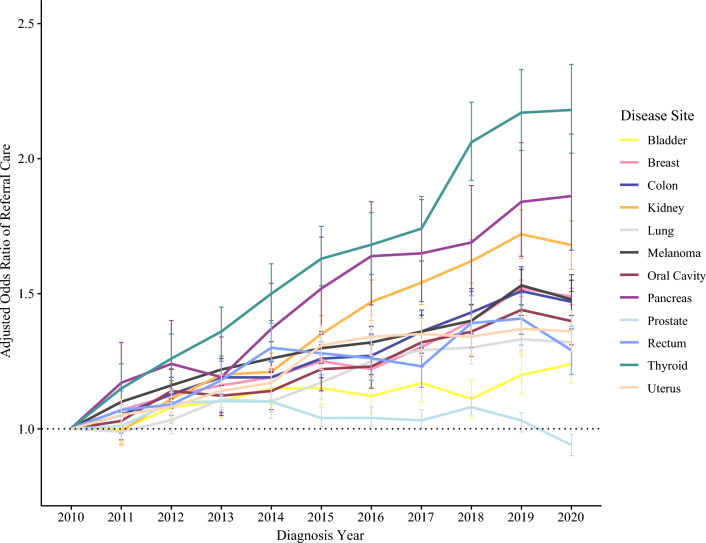
Adjusted odds ratio of referral surgical care by year of diagnosis, grouped by cancer type. Referent year of diagnosis is 2010 for each model; dashed horizontal line indicates an odds ratio of 1.0, and error bars represent 95% confidence intervals. Each line represents a separate multivariable logistic regression model by disease site, adjusted for patient age, race/ethnicity, insurance status, travel distance, metro/urban/rural status, clinical stage, Charlson–Deyo comorbidity index, and CoC facility type

Mixed-effects multivariable logistic regression models were created for each disease site to compare the likelihood of receipt of referred surgical care by cancer type (Supplemental Table 2). After adjustment using the same covariates as the overall model, every disease site except for prostate cancer had a statistically significantly higher odds of referred surgical care over the course of the study period relative to the reference year of 2010 (Fig. 3). Similar to the combined model, increasing likelihood of referred surgical care was associated with increasing clinical stage (range of OR 1.12–5.40 for AJCC Stage III, with 9 of 12 disease sites having an increase in OR between Stage I and Stage III groups; referent Stage 0), and increasing travel distance category (OR range 1.66–3.27 for 25–100 miles, and OR 2.16–5.68 for > 100 miles; referent < 25 miles). Decreasing likelihood of referral surgical care was associated with Non-Hispanic Black race/ethnicity (OR range 0.60–0.90; referent Non-Hispanic White), lack of insurance (OR range 0.46–0.89; referent Private), higher CDCI score (12 of 12 disease sites with decrease in OR from CDCI 1 to CDCI 3 or greater), treatment at CCCP or CCP facilities (statistically significant across both facility types for 10 of 12 disease sites; referent Academic), or moderate- or low-volume facilities (OR < 1.00 for 11 of 12 sites; referent high volume). The ICC values for the facility-level random effects in these disease-specific models ranged from 0.18 (kidney, lung, and pancreas models) to 0.41 (prostate model), with 9 of 12 disease site models having an ICC of 0.20 or greater.

## Discussion

In this study of more than 5 million patients who underwent surgical resection of the 12 most common cancers over a contemporary 10-year period, significant variation in the receipt of referred surgical care and location of surgery, including facility type and volume tertile were seen based on disease site, with certain cancers such as melanoma, uterus, prostate, and oral cavity having high proportions of referred care and treatment at Academic and/or high-volume centers. Furthermore, specific patient and disease characteristics including Medicaid or uninsured status, Non-Hispanic Black race/ethnicity, lower clinical stage, and higher comorbidity burden were associated with decreased likelihood of referred care.

The location where patients receive cancer care influences their receipt of guideline-concordant treatment and clinical outcomes including overall survival, as previously demonstrated across multiple disease sites including breast, colon and rectal, melanoma, prostate, and thyroid cancers, as well as for complex cancer resections.^12–27^ Collectively, these studies and many others have shown that patients undergoing cancer surgery have different outcomes based on the type and/or volume of the facility at which they undergo surgery. Following numerous studies describing the volume-outcome relationships for complex cancer surgery, such as esophagectomy, gastrectomy, and pancreaticoduodenectomy, centralization of care for these cancers through referral to high-volume centers specialized in complex cancer care has increased significantly.^28–31^ Concomitant referral of other surgical care volume, including for more common cancers, has followed these practices for higher-risk surgical cancer care.^32^

While the clinical advantage of centralized care for high-risk surgery are fairly intuitive given the high-intensity resources and specialized personnel needed, advantage for lower-risk cancer surgeries, some of which usually take place in an outpatient setting, are less obvious. Previous work has demonstrated variable clinical benefit for centralization of common cancer care delivery, including bladder, colon, rectal, and lung cancers.^31,33–36^ Sheetz et al. showed in a cohort of Medicare beneficiaries that greater centralization was associated with decreased postoperative complications and death for lung cancer resection, and Pekala et al. also showed decreasing bladder cancer-specific mortality over time alongside increasing centralization of surgical bladder cancer care in a SEER-Medicare cohort.^31,34^ However, Logan et al. demonstrated a decreasing likelihood of adjuvant chemotherapy use in lung cancer patients with increasing travel distance to surgical treatment in an NCDB cohort of patients with early-stage non-small cell lung cancer, suggesting that centralization can have real-world consequences that may affect downstream treatments and outcomes.^35^ Findings from our study support the significant variability of degree of care centralization based on disease site, with a range of 14% to 58% of cancer cases that were referred for surgery.

Interestingly, resection of certain cancers that would traditionally be considered lower-risk surgical cases such as melanoma, thyroid, and uterine cancers were noted to frequently occur in referral settings, most frequently at high-volume Academic facilities. For some disease sites, such as melanoma, uterine, and potentially breast, some portion of this referred surgical care may be explained by the fact that these cancers are frequently diagnosed in an outpatient setting, although this pattern is not applicable for all disease sites studied in this cohort. Overall, these trends in referred surgical care suggest that there may be opportunities for optimization of case allocation among facilities within the same health system if high-quality surgical cancer care can be offered across all sites (i.e. reallocating lower-risk cancer surgeries to relatively lower-volume community centers to potentially increase the availability of surgical cancer care in local communities), while still potentially benefiting from access to specialist expertise within a health system to assist with multidisciplinary treatment planning.

Other potential disadvantages of centralization include increases in travel burden for cancer patients and their caregivers and the risk for exacerbation of underlying healthcare disparities.^2,37,38^ These considerations are important in the context of expanding cancer care expenditures in the United States and the significant contribution that surgical care has to these expenditures, given that resection at high-volume centers may not provide clinically meaningful benefit for all cancer types or stages.^36,39,40^ Although we were unable to quantify travel-associated costs in this retrospective database study, receipt of referred surgical cancer care was associated with significantly longer travel distances compared to combined diagnosis and treatment at the same facility. Additionally, this study found decreased likelihood of referred surgical care for uninsured or Medicaid-insured patients, supporting the impact of individual social risk factors on access to cancer care that has been previously described across many cancer settings.^41–43^ Beyond the difficulties that patients may face in travelling to referral centers or having their referral care covered by insurers, delays in care related to the timely transfer of records and second-opinion pathology or radiology reviews can also impact patients referred for their surgical cancer care.

Another highly relevant issue facing cancer care delivery in the setting of increasing centralization of cancer care is equitable and accessible care for rural populations. While barriers for rural patients can vary based on regional differences, these patients often face unique challenges to accessing health care and particularly subspecialty care.^44–47^ Findings from this study suggest that rural patients were more likely to receive referred surgical care than their metropolitan counterparts after adjusting for other patient and disease characteristics, which may speak to the barriers faced by this population in accessing care closer to their area of residence.

There are several limitations to this study. Importantly, the NCDB does not capture cancer care at non-CoC-accredited facilities. While the NCDB includes more than 70% of newly diagnosed cancers in the United States among the greater than 1500 cancer centers that are accredited by the CoC, nonrandom differences in CoC versus non-CoC hospitals likely exist that would affect the distribution of referred surgical cancer care at these facilities. We suspect that this NCDB cohort may skew toward an overrepresentation of referred care relative to the entire U.S. population but were unable to study non-CoC patients with this dataset, and therefore, these results may not be generalizable to non-CoC facilities. Facility-level variables were only available for the reporting facility; therefore, we were unable to evaluate characteristics of diagnosing facilities to describe what types of facilities are referring cases for surgical resection. The NCDB also suppresses facility type data for patients younger than age 40, which limits our interpretation of these results for disease sites that include higher proportions of younger patients, most notably thyroid cancer. Additionally, facility volume tertiles were study-specific, and development of widely accepted facility-level volume categories for different cancer types in future work may enable better comparison across studies. Finally, the last year of this study was 2020; therefore, these patients’ cancer care including referral care and choice of location of surgery may have been affected by the COVID-19 pandemic. Some patients may have prioritized seeking care closer to their residence due to perceived risk of COVID exposure, whereas hospitals may have enacted varying policies for accepting referral cases and/or allowing elective cancer operations to proceed during early stages of the pandemic, although these effects were not quantifiable in this dataset.

## Conclusions

Significant variation in the receipt of referred surgical cancer and distribution of location of surgery based on disease site as well as patient- and facility-level characteristics were observed in this study of more than 5 million patients with common cancers. These findings provide a contemporary description of the landscape for surgical cancer care in the United States using a hospital-based national dataset that captures the majority of newly diagnosed solid tumors in this country. Future work will evaluate the impact of referral care, facility characteristics, and health system membership on the delivery of guideline-concordant care to better understand how surgical cancer care allocation impacts clinical outcomes for patients with these common cancers.

## Supplementary Information

Below is the link to the electronic supplementary material.

Supplementary file1 (DOCX 16 KB)

Supplementary file2 (DOCX 42 KB)

## Data Availability

The data that support the findings of this study are available from the National Cancer Database. Restrictions apply to the availability of these data, which were used under license for this study. The data are not publicly available due to privacy or ethical restrictions.
