# Supplementary material for: Patterns of Referral for Common Cancer Surgery in the United States
Source: Ann Surg Oncol. Author manuscript; Available in PMC 2025 May 1. (PMC11976353; doi:10.1245/s10434-025-17026-0)
Supplement: Supplemental Table 1 [file NIHMS2059739-supplement-Supplemental_Table_1.docx]

**Supplemental Table 1.** Cohort derivation characteristics from the National Cancer Database (all cohorts 2010-2020).

| **Analytic Disease Group** | **Participant User File** | **ICD-O-3 Histology Codes** | **Primary Site Codes** |
| --- | --- | --- | --- |
| Bladder | Urinary Bladder | 8000-8700, 8720-8790 | C670-C679 |
| Breast | Breast | 8000-8700, 8720-8790, 8982-8983 | C500-C509 |
| Colon | Colon | 8000-8149, 8154, 8160-8231, 8243-8248, 8250-8682, 8690-8700, 8720-8790 | C180-C189, C260 |
| Kidney & Renal Pelvis | Kidney and Renal Pelvis | 8000-8700, 8720-8790 | C649, C659 |
| Lung | Lung, Bronchus NSC | 8000-8700, 8720-8790, 8972, 8980 | C340-C349 |
| Melanoma | Melanoma of the Skin | 8720-8790 | C440-C449 |
| Oral Cavity & Pharynx | Floor of Mouth | 8000-8700, 8982 | C040-C049 |
|  | Gum and Other Mouth | 8000-8700, 8982 | C030-C039, C050-C059, C060-C069 |
|  | Hypopharynx | 8000-8700 | C129, C130-139 |
|  | Lip | 8000-8040, 8042-8180, 8191-8246, 8248-8700, 8982 | C000-C009 |
|  | Major Salivary Glands | 8000-8700, 8720-8790, 8941, 8974, 8980, 8982 | C079-C089 |
|  | Nasopharynx | 8000-8700 | C110-C119 |
|  | Oropharynx (p16-) [includes tonsil] | 8000-8700 | C100-C109 |
|  | Oropharynx HPV-Mediated (p16+) | 8000-8700 | C100-C109 |
|  | Pharynx Other | 8000-8700 | C140, C142, C148 |
|  | Tongue Anterior | 8000-8700, 8982 | C019-C029 |
| Pancreas | Pancreas | ­­8000-8149, 8154, 8160-8231, 8243-8248, 8250-8271, 8272, 8273-8682, 8690-8700, 8720-8790, 8971 | C205-C259 |
| Prostate | Prostate | 8000-8700, 8720-8790 | C619 |
| Rectum & Rectosigmoid Junction | Rectum | 8000-8149, 8154, 8160-8231, 8243-8248, 8250-8682, 8690-8700, 8720-8790 | C209 |
|  | Rectosigmoid Junction | 8000-8149, 8154, 8160-8231, 8243-8248, 8250-8682, 8690-8700, 8720-8790 | C199 |
| Thyroid | Thyroid | 8000-8420, 8430, 8440-8510, 8512-8700, 8720-8790 | C739 |
| Uterus | Corpus Uteri | 8000-8700, 8710-8714, 8720-8790, 8800-8941, 8950-8976, 8980-9111, 9120-9138, 9141-9582 | C540-C549 |
