# Supplementary material for: Patterns of Referral for Common Cancer Surgery in the United States
Source: Ann Surg Oncol. Author manuscript; Available in PMC 2025 May 1. (PMC11976353; doi:10.1245/s10434-025-17026-0)
Supplement: Supplemental Table 2 [file NIHMS2059739-supplement-Supplemental_Table_2.docx]

**Supplemental Table 2A.** Multivariable logistic regression models for each disease site for the likelihood of referred surgical care. CCCP = Comprehensive Community Cancer Program; CCP = Community Cancer Program; INCP = Integrated Network Cancer Program; CDCI = Charlson-Deyo Comorbidity Index; AJCC = American Joint Committee on Cancer.

|  | Bladder | | | Breast | | | Colon | | | Kidney | | | Lung | | | Melanoma | | |
| --- | --- | --- | --- | --- | --- | --- | --- | --- | --- | --- | --- | --- | --- | --- | --- | --- | --- | --- |
| **Characteristic** | **OR** | **95% CI** | **p-value** | **OR** | **95% CI** | **p-value** | **OR** | **95% CI** | **p-value** | **OR** | **95% CI** | **p-value** | **OR** | **95% CI** | **p-value** | **OR** | **95% CI** | **p-value** |
| Age (years) | 0.98 | 0.98, 0.99 | <0.001 | 0.99 | 0.99, 0.99 | <0.001 | 0.99 | 0.98, 0.99 | <0.001 | 1.00 | 1.00, 1.00 | 0.019 | 1.00 | 1.00, 1.00 | <0.001 | 1.00 | 1.00, 1.00 | 0.4 |
| Facility Type |  |  |  |  |  |  |  |  |  |  |  |  |  |  |  |  |  |  |
| Academic | — | — |  | — | — |  | — | — |  | — | — |  | — | — |  | — | — |  |
| CCCP | 0.40 | 0.33, 0.48 | <0.001 | 0.63 | 0.52, 0.77 | <0.001 | 0.68 | 0.58, 0.81 | <0.001 | 0.70 | 0.60, 0.82 | <0.001 | 0.68 | 0.59, 0.80 | <0.001 | 1.08 | 0.83, 1.39 | 0.6 |
| CCP | 0.45 | 0.34, 0.59 | <0.001 | 0.63 | 0.48, 0.84 | 0.001 | 0.67 | 0.52, 0.85 | 0.001 | 0.78 | 0.61, 0.99 | 0.039 | 0.71 | 0.56, 0.92 | 0.009 | 1.31 | 0.91, 1.88 | 0.14 |
| INCP | 0.51 | 0.41, 0.63 | <0.001 | 0.91 | 0.73, 1.14 | 0.4 | 0.86 | 0.71, 1.04 | 0.11 | 1.07 | 0.90, 1.28 | 0.4 | 0.99 | 0.83, 1.18 | 0.9 | 1.25 | 0.94, 1.67 | 0.13 |
| Facility Volume Tertile |  |  |  |  |  |  |  |  |  |  |  |  |  |  |  |  |  |  |
| High | — | — |  | — | — |  | — | — |  | — | — |  | — | — |  | — | — |  |
| Moderate | 0.54 | 0.46, 0.62 | <0.001 | 0.66 | 0.56, 0.78 | <0.001 | 0.59 | 0.52, 0.68 | <0.001 | 0.63 | 0.56, 0.72 | <0.001 | 0.65 | 0.57, 0.74 | <0.001 | 0.63 | 0.51, 0.78 | <0.001 |
| Low | 0.41 | 0.33, 0.51 | <0.001 | 0.55 | 0.44, 0.69 | <0.001 | 0.38 | 0.31, 0.47 | <0.001 | 0.54 | 0.44, 0.65 | <0.001 | 0.66 | 0.54, 0.81 | <0.001 | 0.40 | 0.30, 0.53 | <0.001 |
| Race/Ethnicity |  |  |  |  |  |  |  |  |  |  |  |  |  |  |  |  |  |  |
| Non-Hispanic White | — | — |  | — | — |  | — | — |  | — | — |  | — | — |  | — | — |  |
| Asian/Pacific Islander | 1.06 | 0.97, 1.17 | 0.2 | 1.01 | 0.99, 1.04 | 0.2 | 1.23 | 1.15, 1.32 | <0.001 | 1.09 | 1.02, 1.18 | 0.016 | 1.18 | 1.11, 1.25 | <0.001 | 1.17 | 0.98, 1.39 | 0.090 |
| Hispanic | 0.99 | 0.92, 1.06 | 0.8 | 1.04 | 1.02, 1.06 | <0.001 | 0.99 | 0.94, 1.05 | 0.8 | 1.00 | 0.96, 1.04 | >0.9 | 1.02 | 0.96, 1.08 | 0.5 | 1.01 | 0.93, 1.09 | 0.8 |
| Non-Hispanic Black | 0.75 | 0.71, 0.79 | <0.001 | 0.84 | 0.83, 0.86 | <0.001 | 0.73 | 0.70, 0.76 | <0.001 | 0.81 | 0.78, 0.84 | <0.001 | 0.86 | 0.83, 0.90 | <0.001 | 0.75 | 0.66, 0.85 | <0.001 |
| Other or Unknown | 1.13 | 0.98, 1.30 | 0.081 | 1.20 | 1.15, 1.26 | <0.001 | 1.19 | 1.05, 1.36 | 0.008 | 1.11 | 1.01, 1.23 | 0.035 | 1.22 | 1.08, 1.37 | <0.001 | 1.17 | 1.02, 1.35 | 0.025 |
| Insurance Payor |  |  |  |  |  |  |  |  |  |  |  |  |  |  |  |  |  |  |
| Private Insurance | — | — |  | — | — |  | — | — |  | — | — |  | — | — |  | — | — |  |
| Medicaid | 0.79 | 0.74, 0.85 | <0.001 | 1.01 | 1.00, 1.03 | 0.2 | 0.67 | 0.63, 0.71 | <0.001 | 0.98 | 0.94, 1.03 | 0.4 | 1.04 | 1.00, 1.09 | 0.078 | 0.72 | 0.68, 0.77 | <0.001 |
| Medicare | 1.02 | 0.99, 1.05 | 0.3 | 0.99 | 0.98, 1.00 | 0.076 | 0.95 | 0.91, 0.98 | 0.001 | 0.94 | 0.91, 0.97 | <0.001 | 0.97 | 0.95, 1.00 | 0.053 | 0.98 | 0.95, 1.00 | 0.071 |
| Not Insured | 0.60 | 0.55, 0.67 | <0.001 | 0.84 | 0.81, 0.87 | <0.001 | 0.46 | 0.42, 0.50 | <0.001 | 0.82 | 0.76, 0.88 | <0.001 | 0.89 | 0.81, 0.97 | 0.010 | 0.72 | 0.67, 0.78 | <0.001 |
| Other Government | 1.44 | 1.30, 1.59 | <0.001 | 1.20 | 1.15, 1.25 | <0.001 | 1.16 | 1.02, 1.31 | 0.021 | 1.47 | 1.36, 1.59 | <0.001 | 1.57 | 1.45, 1.70 | <0.001 | 1.62 | 1.50, 1.76 | <0.001 |
| Unknown | 1.01 | 0.90, 1.14 | 0.8 | 1.24 | 1.19, 1.29 | <0.001 | 0.81 | 0.72, 0.91 | <0.001 | 1.19 | 1.09, 1.31 | <0.001 | 1.05 | 0.94, 1.16 | 0.4 | 1.04 | 0.96, 1.13 | 0.3 |
| CDCI Score |  |  |  |  |  |  |  |  |  |  |  |  |  |  |  |  |  |  |
| 0 | — | — |  | — | — |  | — | — |  | — | — |  | — | — |  | — | — |  |
| 1 | 1.00 | 0.97, 1.03 | >0.9 | 0.99 | 0.98, 1.01 | 0.4 | 0.93 | 0.90, 0.96 | <0.001 | 1.01 | 0.98, 1.03 | 0.6 | 1.06 | 1.03, 1.08 | <0.001 | 1.03 | 1.00, 1.06 | 0.077 |
| 2 | 0.94 | 0.89, 0.98 | 0.008 | 0.95 | 0.93, 0.97 | <0.001 | 0.82 | 0.78, 0.87 | <0.001 | 0.97 | 0.93, 1.01 | 0.14 | 1.01 | 0.97, 1.04 | 0.7 | 0.88 | 0.83, 0.92 | <0.001 |
| >= 3 | 0.83 | 0.78, 0.88 | <0.001 | 0.87 | 0.84, 0.91 | <0.001 | 0.67 | 0.62, 0.72 | <0.001 | 0.89 | 0.85, 0.94 | <0.001 | 0.94 | 0.90, 0.98 | 0.005 | 0.82 | 0.76, 0.87 | <0.001 |
| AJCC Clinical Stage |  |  |  |  |  |  |  |  |  |  |  |  |  |  |  |  |  |  |
| 0 | — | — |  | — | — |  | — | — |  | — | — |  | — | — |  | — | — |  |
| 1 | 1.90 | 1.84, 1.96 | <0.001 | 1.35 | 1.34, 1.37 | <0.001 | 1.48 | 1.41, 1.56 | <0.001 | 0.92 | 0.85, 1.00 | 0.039 | 1.64 | 1.43, 1.89 | <0.001 | 1.67 | 1.64, 1.71 | <0.001 |
| 2 | 3.93 | 3.80, 4.06 | <0.001 | 1.58 | 1.56, 1.60 | <0.001 | 1.08 | 1.03, 1.14 | 0.003 | 1.19 | 1.09, 1.29 | <0.001 | 2.45 | 2.13, 2.82 | <0.001 | 2.28 | 2.22, 2.35 | <0.001 |
| 3 | 4.32 | 4.08, 4.57 | <0.001 | 1.64 | 1.61, 1.68 | <0.001 | 1.12 | 1.06, 1.18 | <0.001 | 1.30 | 1.19, 1.41 | <0.001 | 2.72 | 2.36, 3.13 | <0.001 | 1.83 | 1.72, 1.94 | <0.001 |
| 4 | 2.67 | 2.52, 2.82 | <0.001 |  |  |  | 1.05 | 1.00, 1.11 | 0.048 | 2.06 | 1.89, 2.24 | <0.001 |  |  |  |  |  |  |
| Travel Distance |  |  |  |  |  |  |  |  |  |  |  |  |  |  |  |  |  |  |
| <25 miles | — | — |  | — | — |  | — | — |  | — | — |  | — | — |  | — | — |  |
| 25-100 miles | 2.48 | 2.40, 2.57 | <0.001 | 2.50 | 2.47, 2.54 | <0.001 | 2.47 | 2.38, 2.57 | <0.001 | 1.95 | 1.90, 2.00 | <0.001 | 2.39 | 2.33, 2.45 | <0.001 | 2.24 | 2.19, 2.30 | <0.001 |
| >100 miles | 3.95 | 3.72, 4.20 | <0.001 | 4.10 | 3.97, 4.24 | <0.001 | 3.26 | 3.00, 3.54 | <0.001 | 2.16 | 2.06, 2.26 | <0.001 | 3.21 | 3.04, 3.38 | <0.001 | 3.44 | 3.25, 3.64 | <0.001 |
| Urban/Rural Status |  |  |  |  |  |  |  |  |  |  |  |  |  |  |  |  |  |  |
| Metro | — | — |  | — | — |  | — | — |  | — | — |  | — | — |  | — | — |  |
| Urban | 1.02 | 0.98, 1.07 | 0.4 | 1.28 | 1.26, 1.31 | <0.001 | 1.15 | 1.10, 1.21 | <0.001 | 1.19 | 1.15, 1.23 | <0.001 | 1.08 | 1.05, 1.12 | <0.001 | 1.12 | 1.08, 1.16 | <0.001 |
| Rural | 0.94 | 0.85, 1.04 | 0.2 | 1.14 | 1.10, 1.19 | <0.001 | 1.18 | 1.06, 1.31 | 0.002 | 1.14 | 1.05, 1.23 | <0.001 | 1.03 | 0.95, 1.11 | 0.5 | 1.08 | 1.00, 1.16 | 0.062 |
| Diagnosis Year |  |  |  |  |  |  |  |  |  |  |  |  |  |  |  |  |  |  |
| 2010 | — | — |  | — | — |  | — | — |  | — | — |  | — | — |  | — | — |  |
| 2011 | 1.00 | 0.95, 1.07 | 0.9 | 1.07 | 1.05, 1.09 | <0.001 | 1.05 | 1.00, 1.10 | 0.048 | 0.99 | 0.94, 1.05 | 0.8 | 0.99 | 0.94, 1.04 | 0.7 | 1.10 | 1.06, 1.15 | <0.001 |
| 2012 | 1.08 | 1.02, 1.15 | 0.007 | 1.12 | 1.10, 1.14 | <0.001 | 1.13 | 1.08, 1.19 | <0.001 | 1.11 | 1.05, 1.17 | <0.001 | 1.03 | 0.98, 1.09 | 0.2 | 1.16 | 1.11, 1.22 | <0.001 |
| 2013 | 1.10 | 1.04, 1.17 | <0.001 | 1.16 | 1.14, 1.19 | <0.001 | 1.19 | 1.13, 1.25 | <0.001 | 1.20 | 1.14, 1.26 | <0.001 | 1.11 | 1.05, 1.16 | <0.001 | 1.22 | 1.16, 1.27 | <0.001 |
| 2014 | 1.15 | 1.09, 1.22 | <0.001 | 1.19 | 1.17, 1.21 | <0.001 | 1.19 | 1.13, 1.25 | <0.001 | 1.21 | 1.15, 1.28 | <0.001 | 1.10 | 1.04, 1.15 | <0.001 | 1.26 | 1.21, 1.32 | <0.001 |
| 2015 | 1.15 | 1.09, 1.22 | <0.001 | 1.25 | 1.22, 1.27 | <0.001 | 1.26 | 1.19, 1.32 | <0.001 | 1.35 | 1.28, 1.42 | <0.001 | 1.17 | 1.11, 1.23 | <0.001 | 1.30 | 1.24, 1.35 | <0.001 |
| 2016 | 1.12 | 1.06, 1.18 | <0.001 | 1.22 | 1.20, 1.24 | <0.001 | 1.27 | 1.20, 1.35 | <0.001 | 1.47 | 1.40, 1.55 | <0.001 | 1.25 | 1.19, 1.31 | <0.001 | 1.32 | 1.26, 1.38 | <0.001 |
| 2017 | 1.17 | 1.10, 1.23 | <0.001 | 1.30 | 1.27, 1.32 | <0.001 | 1.36 | 1.28, 1.44 | <0.001 | 1.54 | 1.46, 1.62 | <0.001 | 1.29 | 1.24, 1.36 | <0.001 | 1.36 | 1.31, 1.42 | <0.001 |
| 2018 | 1.11 | 1.04, 1.18 | 0.001 | 1.40 | 1.37, 1.43 | <0.001 | 1.43 | 1.35, 1.52 | <0.001 | 1.62 | 1.54, 1.70 | <0.001 | 1.30 | 1.24, 1.37 | <0.001 | 1.40 | 1.34, 1.46 | <0.001 |
| 2019 | 1.20 | 1.13, 1.28 | <0.001 | 1.52 | 1.49, 1.55 | <0.001 | 1.51 | 1.42, 1.60 | <0.001 | 1.72 | 1.63, 1.81 | <0.001 | 1.33 | 1.27, 1.40 | <0.001 | 1.53 | 1.46, 1.59 | <0.001 |
| 2020 | 1.24 | 1.17, 1.32 | <0.001 | 1.49 | 1.46, 1.52 | <0.001 | 1.47 | 1.37, 1.57 | <0.001 | 1.68 | 1.59, 1.77 | <0.001 | 1.32 | 1.25, 1.38 | <0.001 | 1.48 | 1.42, 1.55 | <0.001 |

**Supplemental Table 2B.** Multivariable logistic regression models for each disease site for the likelihood of referral (i.e. “Treatment Only”) care. CCCP = Comprehensive Community Cancer Program; CCP = Community Cancer Program; INCP = Integrated Network Cancer Program; AJCC = American Joint Committee on Cancer.

|  | Oral Cavity | | | | Pancreas | | | | Prostate | | | | Rectum | | | | Thyroid | | | | Uterus | | |
| --- | --- | --- | --- | --- | --- | --- | --- | --- | --- | --- | --- | --- | --- | --- | --- | --- | --- | --- | --- | --- | --- | --- | --- |
| **Characteristic** | **OR** | **95% CI** | **p-value** | **OR** | | **95% CI** | **p-value** | **OR** | | **95% CI** | **p-value** | **OR** | | **95% CI** | **p-value** | **OR** | | **95% CI** | **p-value** | **OR** | | **95% CI** | **p-value** |
| Age (years) | 1.00 | 1.00, 1.01 | <0.001 | 1.00 | | 1.00, 1.00 | 0.5 | 0.96 | | 0.96, 0.96 | <0.001 | 0.99 | | 0.99, 0.99 | <0.001 | 1.00 | | 0.99, 1.00 | <0.001 | 1.00 | | 1.00, 1.01 | <0.001 |
| Facility Type |  |  |  |  | |  |  |  | |  |  |  | |  |  |  | |  |  |  | |  |  |
| Academic | — | — |  | — | | — |  | — | | — |  | — | | — |  | — | | — |  | — | | — |  |
| CCCP | 0.50 | 0.42, 0.59 | <0.001 | 0.66 | | 0.55, 0.79 | <0.001 | 0.54 | | 0.42, 0.70 | <0.001 | 0.64 | | 0.53, 0.77 | <0.001 | 0.63 | | 0.52, 0.76 | <0.001 | 0.54 | | 0.44, 0.66 | <0.001 |
| CCP | 0.55 | 0.42, 0.73 | <0.001 | 0.75 | | 0.47, 1.20 | 0.2 | 0.59 | | 0.40, 0.85 | 0.005 | 0.58 | | 0.44, 0.77 | <0.001 | 0.70 | | 0.52, 0.94 | 0.019 | 0.44 | | 0.31, 0.62 | <0.001 |
| INCP | 0.67 | 0.56, 0.82 | <0.001 | 0.80 | | 0.64, 0.99 | 0.039 | 0.81 | | 0.60, 1.08 | 0.15 | 0.78 | | 0.64, 0.96 | 0.022 | 0.89 | | 0.72, 1.11 | 0.3 | 0.85 | | 0.67, 1.08 | 0.2 |
| Facility Volume Tertile |  |  |  |  | |  |  |  | |  |  |  | |  |  |  | |  |  |  | |  |  |
| High | — | — |  | — | | — |  | — | | — |  | — | | — |  | — | | — |  | — | | — |  |
| Moderate | 0.48 | 0.38, 0.60 | <0.001 | 1.09 | | 0.75, 1.58 | 0.7 | 0.34 | | 0.25, 0.46 | <0.001 | 0.35 | | 0.28, 0.44 | <0.001 | 0.53 | | 0.42, 0.68 | <0.001 | 0.25 | | 0.19, 0.33 | <0.001 |
| Low | 0.56 | 0.49, 0.65 | <0.001 | 0.79 | | 0.66, 0.96 | 0.015 | 0.58 | | 0.47, 0.72 | <0.001 | 0.56 | | 0.48, 0.65 | <0.001 | 0.63 | | 0.54, 0.74 | <0.001 | 0.41 | | 0.34, 0.48 | <0.001 |
| Race/Ethnicity |  |  |  |  | |  |  |  | |  |  |  | |  |  |  | |  |  |  | |  |  |
| Non-Hispanic White | — | — |  | — | | — |  | — | | — |  | — | | — |  | — | | — |  | — | | — |  |
| Asian/Pacific Islander | 1.06 | 0.97, 1.15 | 0.2 | 0.97 | | 0.84, 1.11 | 0.6 | 1.08 | | 1.02, 1.14 | 0.006 | 1.15 | | 1.07, 1.24 | <0.001 | 1.32 | | 1.24, 1.40 | <0.001 | 1.11 | | 1.04, 1.19 | 0.002 |
| Hispanic | 0.93 | 0.87, 1.00 | 0.052 | 0.89 | | 0.80, 1.00 | 0.042 | 1.01 | | 0.98, 1.05 | 0.5 | 1.08 | | 1.02, 1.15 | 0.011 | 1.10 | | 1.05, 1.16 | <0.001 | 1.17 | | 1.11, 1.23 | <0.001 |
| Non-Hispanic Black | 0.60 | 0.57, 0.64 | <0.001 | 0.89 | | 0.82, 0.96 | 0.004 | 0.84 | | 0.82, 0.87 | <0.001 | 0.71 | | 0.67, 0.75 | <0.001 | 0.60 | | 0.56, 0.64 | <0.001 | 0.90 | | 0.87, 0.94 | <0.001 |
| Other or Unknown | 1.03 | 0.89, 1.21 | 0.7 | 0.89 | | 0.69, 1.15 | 0.4 | 1.17 | | 1.08, 1.27 | <0.001 | 1.01 | | 0.88, 1.16 | 0.9 | 1.09 | | 0.96, 1.23 | 0.2 | 1.22 | | 1.08, 1.38 | 0.002 |
| Insurance Payor |  |  |  |  | |  |  |  | |  |  |  | |  |  |  | |  |  |  | |  |  |
| Private Insurance | — | — |  | — | | — |  | — | | — |  | — | | — |  | — | | — |  | — | | — |  |
| Unknown | 0.99 | 0.87, 1.13 | 0.9 | 1.11 | | 0.88, 1.40 | 0.4 | 1.18 | | 1.09, 1.27 | <0.001 | 0.96 | | 0.84, 1.10 | 0.6 | 1.27 | | 1.12, 1.45 | <0.001 | 0.88 | | 0.78, 1.0 | 0.041 |
| Medicaid | 0.77 | 0.73, 0.81 | <0.001 | 0.80 | | 0.71, 0.89 | <0.001 | 0.78 | | 0.75, 0.82 | <0.001 | 0.74 | | 0.70, 0.79 | <0.001 | 0.93 | | 0.88, 0.99 | 0.026 | 1.01 | | 0.95, 1.06 | 0.8 |
| Medicare | 0.90 | 0.87, 0.94 | <0.001 | 0.94 | | 0.88, 1.00 | 0.056 | 1.08 | | 1.06, 1.10 | <0.001 | 0.98 | | 0.94, 1.02 | 0.4 | 0.96 | | 0.92, 1.00 | 0.055 | 0.96 | | 0.93, 0.99 | 0.008 |
| Not Insured | 0.80 | 0.73, 0.87 | <0.001 | 0.68 | | 0.56, 0.83 | <0.001 | 0.76 | | 0.71, 0.82 | <0.001 | 0.53 | | 0.48, 0.57 | <0.001 | 0.81 | | 0.73, 0.91 | <0.001 | 0.79 | | 0.73, 0.85 | <0.001 |
| Other Government | 1.55 | 1.39, 1.74 | <0.001 | 1.38 | | 1.15, 1.65 | <0.001 | 2.17 | | 2.05, 2.29 | <0.001 | 1.38 | | 1.20, 1.57 | <0.001 | 1.03 | | 0.89, 1.18 | 0.7 | 1.19 | | 1.06, 1.34 | 0.004 |
| Charlson-Deyo Comorbidity Score |  |  |  |  | |  |  |  | |  |  |  | |  |  |  | |  |  |  | |  |  |
| 0 | — | — |  | — | | — |  | — | | — |  | — | | — |  | — | | — |  | — | | — |  |
| 1 | 1.05 | 1.01, 1.09 | 0.008 | 1.00 | | 0.95, 1.06 | >0.9 | 0.94 | | 0.92, 0.96 | <0.001 | 0.96 | | 0.92, 1.00 | 0.028 | 1.00 | | 0.96, 1.04 | 0.9 | 1.00 | | 0.97, 1.03 | 0.8 |
| 2 | 0.94 | 0.89, 1.01 | 0.083 | 0.96 | | 0.87, 1.05 | 0.4 | 0.79 | | 0.75, 0.82 | <0.001 | 0.87 | | 0.81, 0.93 | <0.001 | 0.85 | | 0.78, 0.92 | <0.001 | 0.94 | | 0.88, 0.99 | 0.027 |
| >= 3 | 0.95 | 0.87, 1.03 | 0.2 | 0.95 | | 0.84, 1.07 | 0.4 | 0.66 | | 0.62, 0.70 | <0.001 | 0.69 | | 0.63, 0.75 | <0.001 | 0.96 | | 0.85, 1.08 | 0.5 | 0.85 | | 0.78, 0.93 | <0.001 |
| AJCC Clinical Stage |  |  |  |  | |  |  |  | |  |  |  | |  |  |  | |  |  |  | |  |  |
| 0 | — | — |  | — | | — |  |  | |  |  | — | | — |  |  | |  |  | — | | — |  |
| 1 | 1.36 | 1.27, 1.46 | <0.001 | 2.57 | | 2.16, 3.04 | <0.001 | — | | — |  | 3.43 | | 3.20, 3.68 | <0.001 | — | | — |  | 2.25 | | 1.98, 2.55 | <0.001 |
| 2 | 1.43 | 1.33, 1.54 | <0.001 | 3.20 | | 2.69, 3.79 | <0.001 | 1.23 | | 1.21, 1.25 | <0.001 | 5.19 | | 4.84, 5.58 | <0.001 | 1.03 | | 0.99, 1.08 | 0.14 | 2.12 | | 1.85, 2.44 | <0.001 |
| 3 | 1.44 | 1.33, 1.55 | <0.001 | 4.60 | | 3.80, 5.56 | <0.001 | 1.20 | | 1.15, 1.24 | <0.001 | 5.40 | | 5.04, 5.80 | <0.001 | 1.17 | | 1.11, 1.23 | <0.001 | 1.80 | | 1.58, 2.06 | <0.001 |
| 4 | 1.65 | 1.54, 1.78 | <0.001 | 3.55 | | 2.90, 4.36 | <0.001 |  | |  |  | 4.17 | | 3.85, 4.52 | <0.001 | 2.02 | | 1.90, 2.14 | <0.001 |  | |  |  |
| Travel Distance |  |  |  |  | |  |  |  | |  |  |  | |  |  |  | |  |  |  | |  |  |
| <25 miles | — | — |  | — | | — |  | — | | — |  | — | | — |  | — | | — |  | — | | — |  |
| 25-100 miles | 1.98 | 1.91, 2.06 | <0.001 | 1.72 | | 1.62, 1.82 | <0.001 | 2.73 | | 2.67, 2.79 | <0.001 | 3.21 | | 3.07, 3.35 | <0.001 | 1.66 | | 1.60, 1.73 | <0.001 | 3.27 | | 3.16, 3.39 | <0.001 |
| >100 miles | 3.41 | 3.19, 3.64 | <0.001 | 2.51 | | 2.30, 2.74 | <0.001 | 5.68 | | 5.46, 5.92 | <0.001 | 5.30 | | 4.83, 5.81 | <0.001 | 2.47 | | 2.28, 2.69 | <0.001 | 5.22 | | 4.84, 5.64 | <0.001 |
| Urban/Rural Status |  |  |  |  | |  |  |  | |  |  |  | |  |  |  | |  |  |  | |  |  |
| Metro | — | — |  | — | | — |  | — | | — |  | — | | — |  | — | | — |  | — | | — |  |
| Urban | 0.93 | 0.89, 0.98 | 0.004 | 0.88 | | 0.82, 0.95 | 0.002 | 1.08 | | 1.05, 1.12 | <0.001 | 1.21 | | 1.14, 1.28 | <0.001 | 0.97 | | 0.92, 1.03 | 0.4 | 1.19 | | 1.13, 1.24 | <0.001 |
| Rural | 0.89 | 0.79, 0.99 | 0.033 | 0.98 | | 0.81, 1.18 | 0.8 | 1.03 | | 0.97, 1.10 | 0.3 | 1.18 | | 1.05, 1.32 | 0.006 | 0.84 | | 0.73, 0.97 | 0.019 | 1.05 | | 0.94, 1.17 | 0.4 |
| Diagnosis Year |  |  |  |  | |  |  |  | |  |  |  | |  |  |  | |  |  |  | |  |  |
| 2010 | — | — |  | — | | — |  | — | | — |  | — | | — |  | — | | — |  | — | | — |  |
| 2011 | 1.03 | 0.96, 1.10 | 0.4 | 1.17 | | 1.04, 1.32 | 0.010 | 1.01 | | 0.98, 1.05 | 0.4 | 1.07 | | 1.00, 1.14 | 0.043 | 1.15 | | 1.07, 1.24 | <0.001 | 1.05 | | 1.00, 1.11 | 0.064 |
| 2012 | 1.14 | 1.06, 1.22 | <0.001 | 1.24 | | 1.10, 1.40 | <0.001 | 1.10 | | 1.06, 1.14 | <0.001 | 1.09 | | 1.02, 1.16 | 0.011 | 1.26 | | 1.18, 1.35 | <0.001 | 1.08 | | 1.03, 1.14 | 0.003 |
| 2013 | 1.12 | 1.05, 1.20 | <0.001 | 1.19 | | 1.06, 1.34 | 0.004 | 1.10 | | 1.07, 1.14 | <0.001 | 1.18 | | 1.11, 1.26 | <0.001 | 1.36 | | 1.27, 1.45 | <0.001 | 1.14 | | 1.08, 1.20 | <0.001 |
| 2014 | 1.14 | 1.07, 1.22 | <0.001 | 1.37 | | 1.22, 1.54 | <0.001 | 1.10 | | 1.06, 1.13 | <0.001 | 1.30 | | 1.22, 1.39 | <0.001 | 1.50 | | 1.40, 1.61 | <0.001 | 1.17 | | 1.11, 1.23 | <0.001 |
| 2015 | 1.22 | 1.14, 1.31 | <0.001 | 1.52 | | 1.36, 1.71 | <0.001 | 1.04 | | 1.01, 1.08 | 0.013 | 1.28 | | 1.20, 1.36 | <0.001 | 1.63 | | 1.53, 1.75 | <0.001 | 1.31 | | 1.24, 1.38 | <0.001 |
| 2016 | 1.23 | 1.15, 1.31 | <0.001 | 1.64 | | 1.46, 1.84 | <0.001 | 1.04 | | 1.01, 1.08 | 0.022 | 1.26 | | 1.18, 1.35 | <0.001 | 1.68 | | 1.57, 1.80 | <0.001 | 1.34 | | 1.27, 1.42 | <0.001 |
| 2017 | 1.32 | 1.23, 1.41 | <0.001 | 1.65 | | 1.47, 1.85 | <0.001 | 1.03 | | 1.00, 1.07 | 0.064 | 1.23 | | 1.15, 1.31 | <0.001 | 1.74 | | 1.62, 1.86 | <0.001 | 1.35 | | 1.27, 1.42 | <0.001 |
| 2018 | 1.36 | 1.27, 1.46 | <0.001 | 1.69 | | 1.51, 1.90 | <0.001 | 1.08 | | 1.05, 1.12 | <0.001 | 1.39 | | 1.30, 1.49 | <0.001 | 2.06 | | 1.92, 2.21 | <0.001 | 1.34 | | 1.27, 1.42 | <0.001 |
| 2019 | 1.44 | 1.35, 1.54 | <0.001 | 1.84 | | 1.64, 2.06 | <0.001 | 1.03 | | 0.99, 1.06 | 0.12 | 1.41 | | 1.31, 1.50 | <0.001 | 2.17 | | 2.03, 2.33 | <0.001 | 1.37 | | 1.29, 1.45 | <0.001 |
| 2020 | 1.40 | 1.31, 1.51 | <0.001 | 1.86 | | 1.66, 2.09 | <0.001 | 0.94 | | 0.90, 0.98 | 0.001 | 1.29 | | 1.20, 1.38 | <0.001 | 2.18 | | 2.02, 2.35 | <0.001 | 1.36 | | 1.28, 1.44 | <0.001 |
